# Reviewing the Utility of EUS FNA to Advance Precision Medicine in Pancreatic Cancer

**DOI:** 10.3390/cancers10020035

**Published:** 2018-01-27

**Authors:** William Berry, Joanne Lundy, Daniel Croagh, Brendan J. Jenkins

**Affiliations:** 1 Centre for Innate Immunity and Infectious Diseases, Hudson Institute of Medical Research, 27-31 Wright St, Clayton, VIC 3168, Australia; waber1@student.monash.edu (W.B.); joanne.lundy@monash.edu (J.L.); 2 Department of Surgery, Monash University, Clayton, VIC 3800, Australia; daniel.croagh@monash.edu

**Keywords:** Pancreatic cancer, EUS FNA, precision medicine, molecular oncology

## Abstract

Advanced pancreatic cancer (PC) is an aggressive malignancy with few effective therapeutic options. While the evolution of precision medicine in recent decades has changed the treatment landscape in many cancers, at present no targeted therapies are used in the routine management of PC. Only a minority of patients with PC present with surgically resectable disease, and in the remainder obtaining high quality biopsy material for both diagnosis and molecular testing can prove challenging. Endoscopic ultrasound-guided fine needle aspiration (EUS FNA) is a widely used diagnostic procedure in PC, and allows tumour sampling in patients with both early and late stage disease. This review will provide an update on the role of EUS FNA as a diagnostic tool, as well as a source of genetic material which can be used both for molecular analysis and for the creation of valuable preclinical disease models. We will also consider relevant clinical applications of EUS FNA in the management of PC, and the path towards bringing precision medicine closer to the clinic in this challenging disease.

## 1. Introduction

**Figure 1:**
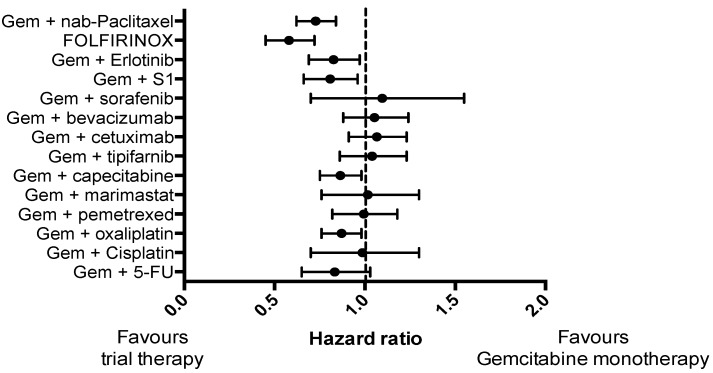
Forest plot demonstrating hazard ratio for phase III clinical trials in pancreatic cancer that included a control arm of gemcitabine monotherapy for a consistent comparison

Pancreatic cancer (PC) is a highly lethal malignancy and ranks as the fourth most common cause of cancer-related death worldwide [1,2]. The prognosis is dire, with a five-year-survival rate of just 5% [3,4]. This is partly because PC is often diagnosed at an advanced stage, with only 20% of patients being suitable for surgical resection [5], and partly due to the fact that PC is largely refractory to non-surgical treatments. Although several treatments have reached Phase II/III clinical trials in advanced PC (Figure 1), only two trials have demonstrated a substantial improvement in survival compared to gemcitabine monotherapy: Gemcitabine and nab-paclitaxel [6]; and FOLFIRINOX (a combination of folinic acid, 5-fluorouracil, irinotecan and oxaliplatin) [7]. However, in unselected patients, even these two promising therapies demonstrate an objective response rate of only 30% [6,7].

The last three decades have seen the evolution of precision medicine, which is now applied to many different cancers. Precision medicine involves reserving specific treatments for patients who have tumours with amenable genetic profiles. Examples include: HER2 amplification in breast cancer which is responsive to trastuzumab [8,9]; *BRAF* mutation in melanoma which predicts responsiveness to BRAF/MEK inhibitor therapy [10,11,12]; *KRAS* wild-type tumours in colorectal cancer which are sensitive to anti-epidermal growth factor receptor (EGFR) therapies [13,14] and *EGFR* mutations and *ALK* gene rearrangements in non-small-cell lung cancer, which are sensitive to EGFR and ALK inhibitors, respectively [15,16]. Given the limited clinical benefits seen with current chemotherapy regimens for PC, the prospect of applying precision medicine to the treatment of PC holds great appeal [17].

## 2. Precision Medicine in Pancreatic Cancer

In recent years, the genomic landscape of PC has become increasingly well characterized, leading to a better understanding of the pathogenesis of PC as well as the identification of a number of potential therapeutic targets [18,19,20,21,22,23,24].

An activating somatic mutation of the KRAS gene has long been identified as a critical event occurring in the vast majority of human PCs [19]. An early mutation in KRAS has been implicated in the progression of pre-malignant pancreatic intra-epithelial neoplasia to invasive malignancy [18], and has also been demonstrated to play a vital role in tumour maintenance [20]. Early KRAS mutations are typically followed by the loss of a number of important tumour suppressor genes, most notably *CDKN2A*, *TP53* and *SMAD4* [21]. 

However, recent studies have demonstrated that beyond these common mutations there is a high degree of heterogeneity among PC tumours, especially those of the pancreatic ductal adenocarcinoma type which represents ~90% of all PC, highlighting a significant challenge when considering the application of precision medicine in this disease [22,23,24,25]. A comprehensive genomic analysis of 24 PC patients identified an average of 63 genetic alterations per tumour, and described alterations in 12 core signaling pathways, some of which (e.g., neoangiogenesis, disrupted DNA damage repair) may offer the potential for therapeutic targeting [22].

On a larger scale, a recent multi-stage, genome-wide association study on over 7000 PC patients with over 14,000 control individuals identified numerous susceptibility loci for PC lying in close proximity to a variety of genes, some of which have previously been implicated in oncogenesis (e.g., *BCAR1*, *KLF14*, *PDX1*, *CHEK2*, *TERT*) [23]. Whole-exome sequencing of resected tumour tissue from a smaller cohort of 109 PC patients reported that approximately 5% of cases contained 24 significantly mutated genes, some of which not only provided prognostic value in terms of disease pathology or patient survival (e.g., *KRAS*, *RBM10*), but also identified patients who may respond to targeted therapies (e.g., *BRAF*, *PIK3CA*) [24].

The high genetic diversity of PC tumours provides a potential explanation for the relatively slow progress in the development of novel and effective chemotherapies for PC, especially since new treatments have previously been tested on unselected PC patient populations [6,7,25,26]. Accordingly, personalised therapeutic approaches based on the genetic profile of individual tumours in PC provide the opportunity to vastly improve patient outcomes [17]. Indeed, using resources such as the COSMIC database (Catalogue Of Somatic Mutations In Cancer), we can identify a number of commonly mutated genes that may make that tumour amenable to specific therapy (Table 1), despite many occurring at a low overall frequency [27]. 

Of note, these studies used surgical resection specimens to provide tissue for the isolation of genetic material. This reliance on surgical resection specimens effectively excludes the possibility of real time genetic analysis for many patients with advanced disease. Consequently, one of the major obstacles to the introduction of precision medicine in PC has been the difficulty in recruiting patients with high quality tumour-derived genetic material (genomic DNA and/or RNA) in sufficient quantities for subsequent molecular profiling, as recently reported by the Individualized Molecular Pancreatic Cancer Therapy (IMPaCT) Trial. The IMPaCT trial was designed to identify subsets of patients with advanced metastatic disease who could be targeted, based on mutations within their tumour genome, with currently available therapies [33]. A major limitation of this study was the heavy reliance on archival formalin-fixed, paraffin-embedded (FFPE) samples for genomic DNA extraction, most of which were derived from surgical resections which are possible in only 20% of PC patients [5]. Accordingly, there is an urgent and unmet clinical need to improve methodologies for the robust isolation of high quality genetic material in a timely manner from the vast majority of PC. 

## 3. EUS FNA as a Source of Genetic Material

The above observations highlight a potential role for EUS FNA to isolate genetic material to direct precision medicine. Up to 50% of PC patients present with locally advanced disease and undergo EUS FNA to establish a tissue diagnosis. Although EUS FNA has been used to provide tissue for the genetic analysis for PC and other cancers, the clinical utility of this technique has been hampered by concerns about low tissue quantities leading to a suboptimal yield of genetic material, as well as sample contamination with non-malignant cells [46,47,48,49,50]. Nonetheless, the inherent advantage of EUS FNA is the ability to sample locally advanced tumours, which are unsuitable for surgical resection, giving clinicians the ability to obtain tissue which would be otherwise unavailable [5]. EUS FNA is generally considered a safe procedure, and a large systematic review of over 10,000 patients reported reassuringly low morbidity (0.98%) and mortality (0.02%) rates associated with EUS FNA [51].

Indeed, EUS FNA is possible at all stages of disease and can be repeated more easily than other biopsy techniques, further highlighting its potential utility. For example, it could be used to guide selection of neo-adjuvant therapy in operable patients, while in those patients receiving palliative chemotherapy it allows for repeat sampling of the tumour, which might allow assessment of whether a particular therapy is having the desired effect on the specific molecular pathway targeted by the treatment. Here we review the use of EUS FNA as a source of genetic material from PC tumours and examine the potential to use this technique in guiding precision medicine in PC.

A number of groups have examined the use of EUS FNA-derived DNA for the detection of KRAS mutations in order to improve diagnostic sensitivity of EUS FNA [49,50,52,53,54,55,56,57]. KRAS mutations are found in approximately 80–90% of PC, and therefore a sample that has inconclusive cytology but is positive for a KRAS mutation strongly suggests a malignant diagnosis. A recent meta-analysis on the topic pooled eight studies and determined that combining standard EUS FNA cytology with KRAS testing increased the sensitivity of PC diagnosis from 80.6% to 88.7%, and reduced the false-negative rate by 55.6% [48]. It is worth noting that the improvement in diagnostic rate reported in this meta-analysis is comparable to that seen when implementing more convenient methods such as on-site cytology, which also improves the sensitivity of EUS FNA from approximately 80% to 88% [46,48]. A recent study reports that adding selected immunohistochemical markers can increase the diagnostic sensitivity even further to 95% [58]. Together, these studies demonstrate that EUS FNA can be used for the isolation of gDNA and can potentially be used to improve diagnostic accuracy. Other approaches which may improve the diagnostic yield of EUS FNA include the use of 25G or 19G needles instead of the standard 22G needle, adding suction, increasing the number of passes performed, rinsing the EUS needle with sterile saline, and optimizing sample processing [59,60]. The yield of high quality genomic material from biopsies has also been described to be improved with techniques such as snap freezing biopsies in liquid nitrogen, or using agents such as RNA *later^®^* to preserve RNA [61].

Many studies have relied upon formalin-fixed and paraffin-embedded (FFPE) samples for the isolation of gDNA. The amount of FFPE tissue available from EUS FNA is often very limited and the fixation process can result in nucleic acid fragmentation, which can reduce the yield and quality of gDNA and substantially degrades RNA, thereby reducing the potential for this method to detect mutations in tumoural DNA and eliminating the ability to measure gene expression. This suggests that the sensitivity of EUS FNA and KRAS mutations could be further improved if more reliable DNA extraction techniques were used, such as snap freezing of biopsies. 

The added utility of KRAS mutation assay in the diagnosis of cystic lesions within the pancreas has also been examined. Overall, cytological analysis has a much lower sensitivity in cystic lesions of the pancreas compared to solid lesions, only ranging from 35–63% [62,63]. In a recent meta-analysis of eight studies that included KRAS status as a diagnostic adjuvant where identified, the authors found that sensitivity improved from 42% to 71% when molecular analyses were used in conjunction with conventional cytology [64]. The authors acknowledge that these findings may be subject to bias as only patients with a verified diagnosis on surgical resection could be included. However, the same bias would apply to the accuracy of cytology alone, and therefore these findings strongly indicate that the addition of a genetic analysis improves diagnostic accuracy. 

To date, only a limited number of studies have reported the use of EUS FNA-derived RNA in PC, including one study from our institution [65,66,67]. Among these, Rodriguez and colleagues examined whether it was possible to use EUS FNA-derived RNA to diagnose PC in the place of cytology [66]. A total of 48 patients were enrolled in this study, among which samples from nine patients were excluded because of either insufficient RNA yield (n = 6) or a final histopathological diagnosis as neuroendocrine tumours (n = 3). This study used RNAseq to profile malignant and benign samples and generate a gene signature to differentiate these two diagnoses. A training set of 13 patient samples was used to generate a gene signature that differentiated between benign and malignant samples. On a separate set of 23 patients (15 malignant and eight benign), the signature was found to have a sensitivity of 0.87 and a specificity of 0.75. However, this analysis did not include the 20% (9/45) of samples that had insufficient RNA yields to be included in the analysis. Once again, this was a retrospective study and only included patients with a confirmed diagnosis, and therefore the diagnostic value of this gene signature in “inconclusive” cytological diagnoses cannot be assessed. As mentioned previously, cytology alone is highly specific and has a sensitivity approaching 90% when on site cytology is available. Therefore, this study shows that although EUS FNA-derived RNA can be used for meaningful genetic analysis, almost one in five patients were unable to be characterized due to low RNA yields. While the diagnostic sensitivity of EUS FNA-derived RNA was disappointingly low, it is worth noting that attempts to derive a diagnostic gene signature from surgical specimens have also resulted in similar accuracy [68]. This low diagnostic sensitivity may be consequence of the genetic heterogeneity of PC outlined above. 

In an earlier study by Bournet et al. [67], EUS FNA was employed in the context of a prospective study to investigate its clinical feasibility as a technique to profile RNA extracted from locally advanced and/or metastatic pancreatic ductal adenocarcinoma patients, along with control individuals diagnosed with chronic pancreatitis. While this study indicated that RNA could indeed be extracted from patient tumour biopsies for gene expression profiling, among the 108 PC patients enrolled, again only a fraction of samples from these patients (40%; 44/108) were included for analysis due to poor quantity and quality of RNA extracted from the majority of EUS FNA-acquired samples [67]. Furthermore, the clinical utility of this study was limited because expression profiling was only performed using low density arrays comprising 23 candidate genes [67].

Berry et al., attempted to use transcriptome profiling from metastatic and localised PC to determine whether there was a difference in the genetic profiles of these two clinical phenotypes [65]. Interestingly, when RNAseq was performed on tissue isolated from the primary tumour by EUS FNA, there were no differentially expressed genes between 20 localised and 20 metastatic tumours, suggesting that there is little difference in gene expression between these two clinical subtypes of disease. However, it is possible that any gene expression changes between the localized and metastatic phenotypes are being obscured by the extensive inter-tumoural heterogeneity within PC. 

Nevertheless, these studies suggest that highly sensitive sequencing studies are indeed possible with EUS FNA-derived material, and therefore that this technique has the potential to guide precision medicine for PC patients. A recent study attempted to assess the potential for this strategy using the Human Comprehensive Cancer GeneRead™ DNAseq Targeted Panel V2 (Qiagen Inc, Valencia, CA, USA), a panel which detects mutations across the exome (coding regions) of 160 genes frequently mutated in malignancy [69]. Included in this panel are a number of “targetable mutations”, for which there is a specific therapy that has been effectively used in selected patients with other tumour types (e.g., BRAF, BRCA, PALB, ERBB1 and ERBB2). Although the authors report that they did not identify any tumours with “targetable mutations” they neglected two phenotypes for which there are in fact potential treatments. First, KRAS wild-type tumours occurred in 6.9% of patients, and there is some evidence to suggest that such tumours may be amenable to EGFR blockade [14,28,29,70]. Second, an ATM mutation was also identified, and these tumours may be susceptible to DNA-damaging agents [31]. Furthermore, this panel does not allow for copy number variation to be assessed, which might reveal more targets for precision medicine (e.g., HER2 amplification). One of the most striking findings in this study was a comparison of EUS FNA-derived and surgically-derived DNA, which revealed that 83.3% (15/18 patients) had 100% gene mutational concordance, and the allelic frequency of mutations was 34% and 35% in EUS FNA-derived and surgically-derived Dann, respectively. Together, this validates the use of EUS FNA for identifying mutations in PC gDNA and shows that the tumour cell content in both sample types is similar. Ultimately, this study demonstrates that precision medicine based on the mutation status of particular target genes can indeed be directed by EUS FNA-derived gDNA. 

## 4. EUS FNA versus Surgical Specimens

The overwhelming majority of work to characterize the genetic phenotypes of PC has been performed with surgical resection specimens. This makes the interpretation of novel phenotypes identified using EUS FNA problematic. Although it has been demonstrated that allelic frequency is comparable between EUS FNA-derived and surgically-derived DNA, it is unclear what impact the sampling method has on non-tumoural cell content and gene expression. For instance, EUS FNA samples typically contain blood, inflammatory cells and even intestinal wall epithelial cells; whereas, surgical specimens contain large amounts of stromal tissue but no contamination with intestinal wall cells. Consequently, gene expression studies using EUS FNA-derived material need to address these differences. In studies using surgical resection specimens, samples are usually acquired in the presence of the surgeon and pathologist and a frozen section is often performed to quantify the tumour cellularity within the sample. Alternatively, micro-dissection can be performed to maximise tumour cellularity [30,42,71]. These conditions cannot be replicated in the endoscopic environment even with on-site cytology. EUS FNA has a diagnostic sensitivity of only approximately 90%, largely due to sampling error and/or failure to obtain sufficient cells. Furthermore, any attempt to quantify the relative contributions of various cell types in the EUS FNA specimen (for example with fluorescence activated cell sorting) will inevitably lead to a significant reduction in the amount of genetic material recovered from the specimen. These inherent limitations with EUS FNA will have implications for the interpretation of the genetic profile of PC provided by this technique. Conversely, the obvious advantage of EUS FNA is the ability to obtain tissue from all patients with relative ease and at minimal additional cost. 

**Figure 2:**
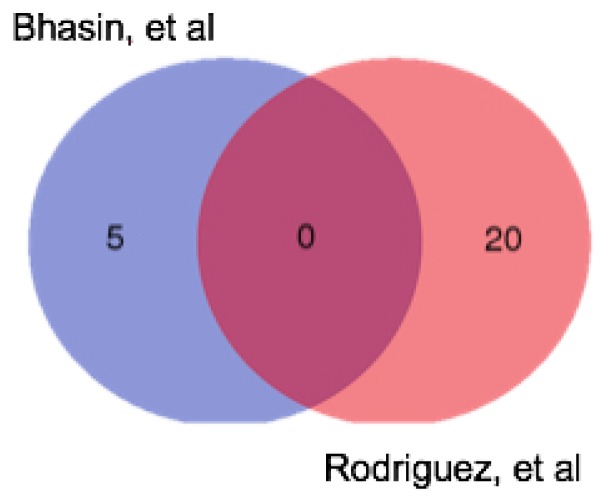
Venn diagram showing the differences between the genes up-regulated in PC in two published diagnostic gene signatures. Bhasin et al. [68] presented a five-gene signature up-regulated in PC compared to normal pancreas; Rodriguez, et al. [66] presented an 83 gene signature of up- and down-regulated in PC, they only published 20 up-regulated genes and 20 down-regulated genes.

We have already alluded to the similarities between the two sample types in terms of mutational concordance, but what of the differences? Diagnostic gene signatures have been used for both resection specimens and EUS FNA samples to distinguish PC from non-malignant tissue (pancreatitis or normal pancreas). Bhasin et al. [68] performed a meta-analysis on 12 microarray studies that contrasted PC with normal pancreas, using RNA obtained from resection specimens. The authors identified a five-gene signature that had a sensitivity and specificity of 95% and 89%, respectively. Comparing these five genes to the list of up-regulated genes generated through similar analyses on EUS FNA-derived RNA by Rodriguez, et al. [66], we observe that no genes were common to both gene lists (Figure 2). This suggests that EUS FNA and surgical samples may indeed reflect two distinct sample types. Interestingly, Moffitt, et al. [72] used transcriptome profiling of tumour samples and adjacent normal tissue to perform a “virtual microdissection” to compare tumours at primary and metastatic sites. In keeping with our observation that the expression profile of localized and metastatic tumours was very similar, they demonstrated that previously reported differences in the transcriptome profiles or primary and metastatic tumours were likely due to contamination with surrounding tissue and that the tumour profiles remain similar despite the anatomical location changing during metastasis. Overall, this suggests that differences seen in the transcriptome profile between EUS FNA and resection specimens may reflect differences in the nature and degree of “contaminating” cells rather than differences in the tumour profile. Although the sampling techniques in both cases may be equivalent in terms of tumour cell content, the degree of contamination with surrounding normal and inflammatory tissue in both cases might alter the profile of the tumour.

## 5. Preclinical Disease Models for Precision Medicine 

In a pre-clinical setting, precision therapy can be tested using patient-derived xenograft (PDX) models or organoid culture for in vivo and in vitro drug testing [73,74,75]. PDX studies involve the implantation of cancer cells obtained from the patient into an immune-deficient mouse, and in the context of immunotherapy provide the advantage over organoids of having the capacity of being ”humanized” in vivo via reconstitution with human immune cells [76]. Over a range of tumour types, including PC, xenografted tumours have been shown to retain the characteristics of the original patient tumour in terms of histological architecture and molecular profiles [77,78,79,80,81,82]. This makes xenograft models valuable tools to demonstrate a biological response to precision therapies designed to target specific tumour molecular profiles [83]. However, models for PC have largely been restricted to utilizing surgical resection specimens. There have only been two reports of the use of EUS FNA samples to create patient-derived xenograft models—one in cholangiocarcinoma and one in PC [65,84]. Both of these studies demonstrate that grafting EUS FNA-derived tissue is indeed viable, however, neither study reports the graft rate (or failure rate), therefore the grafting efficiency of this technique has not been established. In our experience the grafting efficiency is quite low and grafts may take many months to develop, largely due to the small amount of tumour tissue obtained by EUS FNA, which limits the usefulness of this technique to guide real-time therapy selection in the patients from which the PDX were derived.

Recently, the growth of patient-derived tumour tissue in vitro using specifically defined media and conditions (organoid culture) provides a complementary tool to PDXs to test the anti-cancer efficacy of new compounds [74,75,85]. These cultures provide a valuable new pre-clinical model of disease which can be maintained indefinitely while also maintaining genomic stability, and successfully frozen and thawed allowing for long term storage [74,86,87]. Three-dimensional organoid cultures can be generated using tissue from EUS FNA samples with high levels of success in a timeframe of just weeks; can be serially passaged, and used to generate PDX [75]. The rapidity with which these cultures can be established means that they are suitable for detailed cellular and molecular analysis of PC, and allow for longitudinal testing for responsiveness to various therapeutics [74,75,85]. Organoid cultures are therefore poised to also play an important role in the introduction of precision medicine in PC.

## 6. Clinical Applications of Molecular Analysis

There are a number of potential ways to integrate molecular analyses into clinical decision-making, including for the identification of new molecular targets, to guide the selection of therapy, and in monitoring disease. 

The identification of known molecular targets allows for treatment with currently available therapeutics; examples include treating HER2-amplified tumours with trastuzumab or KRAS wild-type tumours with EGFR inhibitors [33]. Other potential therapeutic targets are listed in Table 1. Using this approach, these molecular analyses are well placed to identify new targets for future drug development and to improve upon previous results [30,71]. 

A phase III clinical trial of gemcitabine with or without erlotinib, an EGFR inhibitor, as the first line therapy in unselected patients with locally advanced or metastatic PC demonstrated a statistically significant improvement in overall survival in the combination arm, albeit only in the manner of approximately two weeks [26]. Given that the presence of a KRAS mutation is a negative predictor of response to EGFR directed therapy in colorectal cancer [14,88], the fact that the overwhelming majority of PC cases harbor a KRAS mutation may be a significant factor in the failure of this study to show a more substantial survival benefit. Utilising techniques such as EUS FNA to allow for molecular analysis to select patients with KRAS wild-type tumours for enrolment into prospective clinical trials of EGFR inhibitors is therefore an appealing concept, but one that has so far proved difficult in clinical practice [33].

The use of immunotherapy agents such as antibodies against programmed-death-1 receptor (PD-1) has dramatically improved treatment responses in some malignancies such as advanced melanoma [89], but to date responses to similar agents in PC have been disappointing [90,91]. In colorectal cancer, it has been observed that tumours harbouring mismatch repair deficits carry a greater mutational load and are more likely to respond to immunotherapy [92], suggesting a similar response could be seen in the subset of patients with advanced PC whose tumours harbor similar characteristics. The reported incidence of micro-satellite instability and mismatch repair deficits in PC varies greatly, from just 0.3–3.7% in unselected patient populations [37,38], to 8.6% in selected subgroups of long-term survivors [39], up to 22% in patients with medullary histology [40]. Interestingly, mismatch repair deficits are reported to occur more commonly among patients with a KRAS wild-type phenotype [38]. 

Despite recent advances, translating promising pre-clinical results into meaningful clinical benefits remains elusive, as reflected in the disappointing results of early phase clinical trials utilising novel agents such as hedgehog inhibitors in unselected PC patients [93,94], and mechanistic target of rapamycin (mTOR) inhibitors in patients with tumours with PIK3CA amplification or mutation or PTEN loss [41]. 

At present, chemotherapy remains the standard of care for the management of advanced PC [3], although only a minority of patients will attain an objective response to therapy [6,7]. Identifying subgroups of patients more or less likely to respond to chemotherapy could therefore have significant clinical relevance. A recent example of this concept is the retrospective identification of human equilibrative nucleoside transporter-1 (hENT1) expression as a predictive biomarker in PC patients undergoing gemcitabine-based chemotherapy, in this case primarily obtained through immunohistochemistry and quantitative PCR analysis using previously obtained FFPE surgical samples [58,95,96]. Yamada, et al. reported high concordance between hENT1 expression in baseline EUS FNA samples compared to subsequent surgical specimens in patients receiving neoadjuvant gemcitabine-based chemo–radiotherapy, and confirmed the role of hENT1 in predicting gemcitabine response [97]. This supports the clinical utility of EUS FNA biopsy in treatment naïve patients, to evaluate the molecular profile of tumours and identify novel biomarkers both before and during treatment. 

While treating a specific molecular abnormality with a targeted therapy is appealing, there may be a complex array of interacting genes contributing to treatment susceptibility and resistance mechanisms [19,24,98]. The utilisation of machine learning algorithms can allow researchers to investigate high numbers of potential correlations and obviates the potential for bias when selecting candidate genes [99,100,101]. This process allows a computer to make connections between the outcome (response) and the data (molecular profile) in a training dataset. These connections are then applied to a separate dataset and false discoveries are then eliminated as “quirks” specific to the original training dataset. The adjusted algorithm is finally applied to a validation dataset, which measures the algorithm’s accuracy. This is a way of identifying molecular factors specific to each patient’s tumour that may direct clinicians to deliver more efficacious therapies.

Together, these applications can have an immediate impact on patient outcomes by directing clinicians to use targeted therapies in a personalised manner. In addition, the future implications in terms of drug discovery offer exciting new directions in molecular oncology research.

However, despite recent advances, in clinical practice the translation of promising data from the bench to the bedside remains challenging. The IMPaCT trial, mentioned above, clearly demonstrated some of these hurdles and highlighted the need for flexible, pragmatic study designs for personalised medicine trials in PC [33]. Despite screening over 90 patients for suitably targeted therapy, at the time of reporting no patients had been successfully treated in the study. One major limiting factor in this study was the difficulty obtaining suitable tumour specimens for testing in a timely manner, a critical factor in PC, where the disease course can unfortunately be rapid and the window for therapeutic intervention treatment relatively narrow [2].

## 7. Conclusions

There is a clear unmet clinical need for the advancement of therapeutic options in advanced PC. EUS FNA has only recently emerged as a candidate for isolating genetic material from PC and therefore as a means for biomarker identification, as well as in the establishment of valuable pre-clinical disease models such as PDX and organoid cultures. A major advantage of EUS FNA compared to surgical resection specimens is the ability to include patients with unresectable disease, who make up the overwhelming majority in PC. In addition, the ability to obtain tissue early in the clinical course of the disease facilitates the procurement of chemo-naïve tissue. Given the success in obtaining both gDNA and RNA and translating these into meaningful genomic and transcriptomic data, EUS FNA is poised to identify and characterise known and novel therapeutic biomarkers. As such, new trials of personalised therapy in PC should endeavor to use EUS FNA to direct treatment as well as, or instead of, surgical specimens. By doing so, we may overcome some of the practical barriers which currently limit the clinical application of precision medicine for PC.

## Figures and Tables

**Table 1:** Precision medicine: therapeutic targets in PC.

Target	Treatment	Estimated Prevalence
*KRAS* wild-type	EGFR inhibitors (e.g., panitumumab, cetuximab, erlotinib)	10–20% [24,28,29,30]
DNA repair pathway defects (*BRCA1, BRCA2, PALB2, ATM*)	DNA damaging agents (e.g., mitomycin C, platinums) PARP inhibitors (e.g., olaparib)	4–20% [22,24,30,31]
*HER2* amplification	Anti-HER2 antibodies/tyrosine kinase inhibitors (e.g., trastuzumab/lapatinib)	10–30% [32,33]
*MET* activation (mutation, overexpression, amplification)	MET inhibitors	20% [34,35,36]
Mismatch repair gene deficits (*MLH1, MSH2, MSH6, PMS2*)	Immunotherapy	3–22% [37,38,39,40]
*PIK3CA* amplification/mutation +/− *PTEN* loss	mTOR inhibitors (e.g., everolimus)	15–20% [24,41,42]
*CDKN2A* loss	CDK4/6 inhibitors (e.g., palbociclib)	25% [22,43,44,45]
*BRAF* mutation	BRAF inhibitors (e.g., dabrafinib), MEK inhibitors (e.g., trametinib)	2% [19,24,27,42]
*FGFR1* amplification	FGFR inhibitors	1% [27,30]
